# A Novel Homozygous SCNN1B Variant Causing Severe Systemic Pseudohypoaldosteronism Type 1B in a Saudi Infant: A Case Report

**DOI:** 10.7759/cureus.106241

**Published:** 2026-03-31

**Authors:** Ali Alquraishi, Musa M Saad, Ruba Alqahtani

**Affiliations:** 1 Department of Pediatrics, Armed Forces Hospital Southern Region (AFHSR), Khamis Mushait, SAU; 2 Department of Day Surgery (DSU), Armed Forces Hospital Southern Region (AFHSR), Khamis Mushait, SAU

**Keywords:** consanguinity, epithelial sodium channel (enac), hyperkalemia, novel variant, salt-wasting, scnn1b gene, systemic pseudohypoaldosteronism type 1b

## Abstract

Pseudohypoaldosteronism type 1B (PHA1B) is a rare autosomal recessive disorder characterized by systemic resistance to aldosterone due to pathogenic variants in epithelial sodium channel (ENaC) subunit genes. We report an eight-month-old Saudi male infant, born to consanguineous parents, who initially presented at seven days of life with persistent hyperkalemia, hyponatremia, and metabolic acidosis. Despite aggressive medical management, including sodium supplementation, insulin-dextrose therapy, nebulized salbutamol, and peritoneal dialysis, electrolyte disturbances persisted. Whole-exome sequencing identified a novel homozygous likely pathogenic variant in the SCNN1B gene (NM_000336.2:c.1573C>T; p.Gln525*), confirming the diagnosis of systemic PHA1B.

## Introduction

Pseudohypoaldosteronism type 1 (PHA1) is a rare but potentially life-threatening disorder of electrolyte balance that typically presents in early infancy with salt-wasting, hyperkalemia, metabolic acidosis, and dehydration. It results from resistance to the action of aldosterone, a hormone that normally promotes sodium reabsorption and potassium excretion in the distal nephron [1]. Because of its clinical presentation, PHA1 is frequently mistaken for more common conditions such as congenital adrenal hyperplasia, making early recognition essential.

Two major clinical forms of PHA1 are recognized: the autosomal dominant renal-limited form and the autosomal recessive systemic form [2]. The systemic form is more severe and is caused by pathogenic variants affecting the epithelial sodium channel (ENaC), which plays a critical role in sodium transport across epithelial tissues including the kidney, colon, lungs, and sweat glands [3]. Loss-of-function variants in the genes encoding ENaC subunits (SCNN1A, SCNN1B, and SCNN1G) lead to impaired sodium reabsorption in the distal nephron, resulting in severe neonatal salt-wasting and persistent hyperkalemia [4]. Affected infants often present with life-threatening electrolyte disturbances in the neonatal period and may experience recurrent metabolic crises, particularly during intercurrent illnesses [5].

Advances in molecular diagnostics, particularly next-generation sequencing techniques such as whole-exome sequencing, have improved the identification of pathogenic variants associated with systemic PHA1 and expanded the spectrum of known mutations [6]. Despite this, the condition remains rare, and reports describing novel variants continue to contribute to a better understanding of its clinical and genetic heterogeneity. We report a Saudi infant with severe systemic pseudohypoaldosteronism type 1 caused by a novel homozygous variant in the SCNN1B gene, emphasizing the diagnostic challenges, clinical course, and management complexities associated with this rare condition.

## Case presentation

An eight-month-old Saudi male infant, born to consanguineous parents, was delivered at term via unassisted normal vaginal delivery with a birth weight of 2.6 kg. Antenatal ultrasonography revealed bilateral pelvicalyceal system dilatation. At seven days of age, he was admitted to the neonatal intensive care unit for further evaluation. A micturating cystourethrogram showed no abnormalities.

On admission, the infant appeared well and active. His vital signs were within normal limits, with a heart rate of 122 beats per minute, respiratory rate of 33 breaths per minute, and blood pressure of 75/55 mmHg. He exhibited no dysmorphic features, had normal male genitalia without hyperpigmentation, and his systemic examination was unremarkable.

During his NICU stay, he developed persistent electrolyte abnormalities, including significant hyperkalemia (5.5-8.6 mmol/L), hyponatremia (as low as 119 mmol/L), and mild metabolic acidosis. Congenital adrenal hyperplasia was initially suspected. Management included high-dose sodium supplementation and multiple interventions for hyperkalemia, including intravenous calcium gluconate, nebulized salbutamol, intravenous insulin with 10% dextrose, sodium bicarbonate, and potassium-binding resins. Fludrocortisone was initiated at 0.1 mg daily and escalated to 0.6 mg daily without clinical improvement. The lack of response to mineralocorticoid therapy further supports the diagnosis of aldosterone resistance. Due to refractory hyperkalemia, the patient required peritoneal dialysis. Broad-spectrum antibiotics were administered for suspected sepsis.

**Figure 1:**
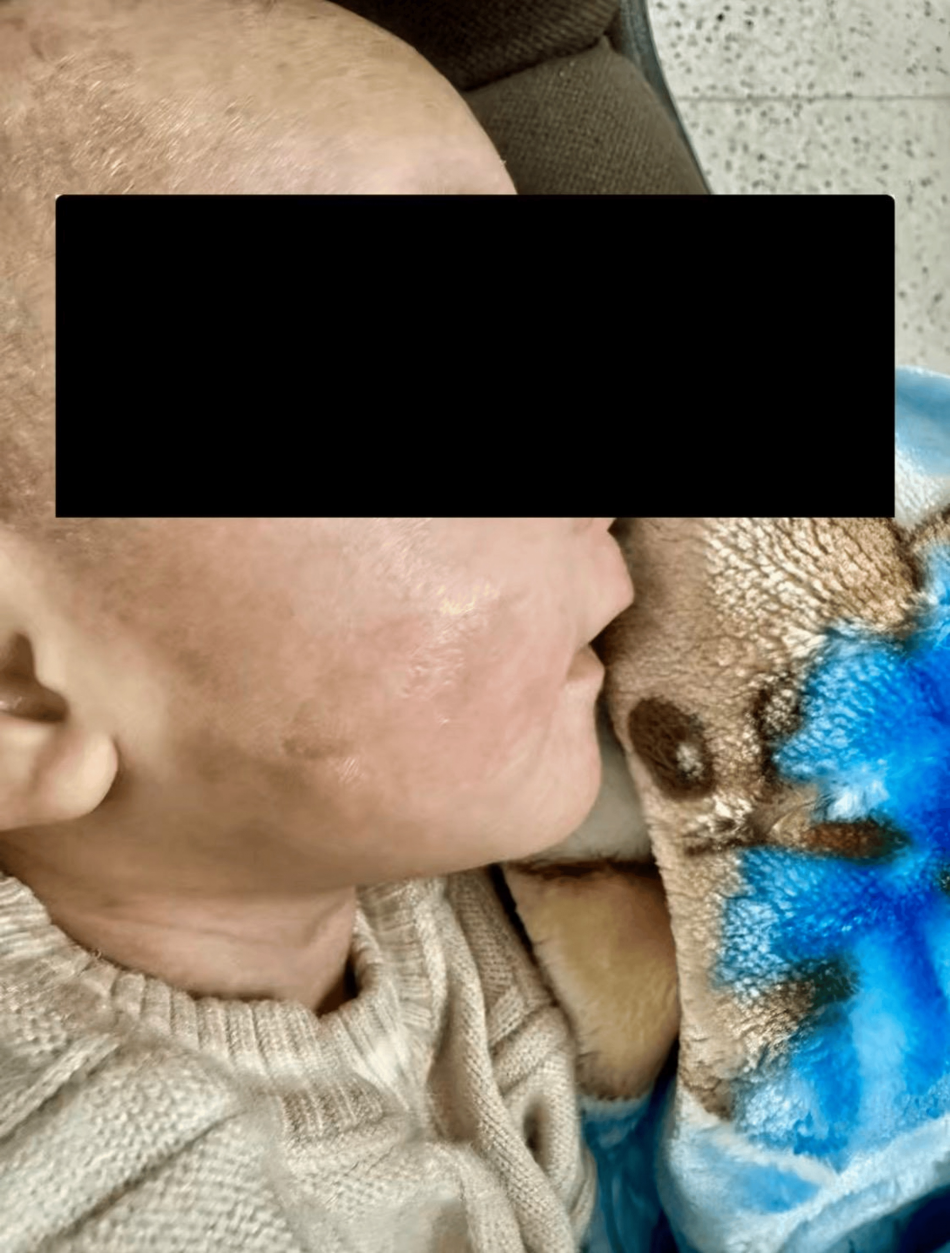
Erythematous dermatitis involving the face and trunk observed during hospitalization, likely related to increased sodium content in sweat in systemic pseudohypoaldosteronism type 1

**Figure 2:**
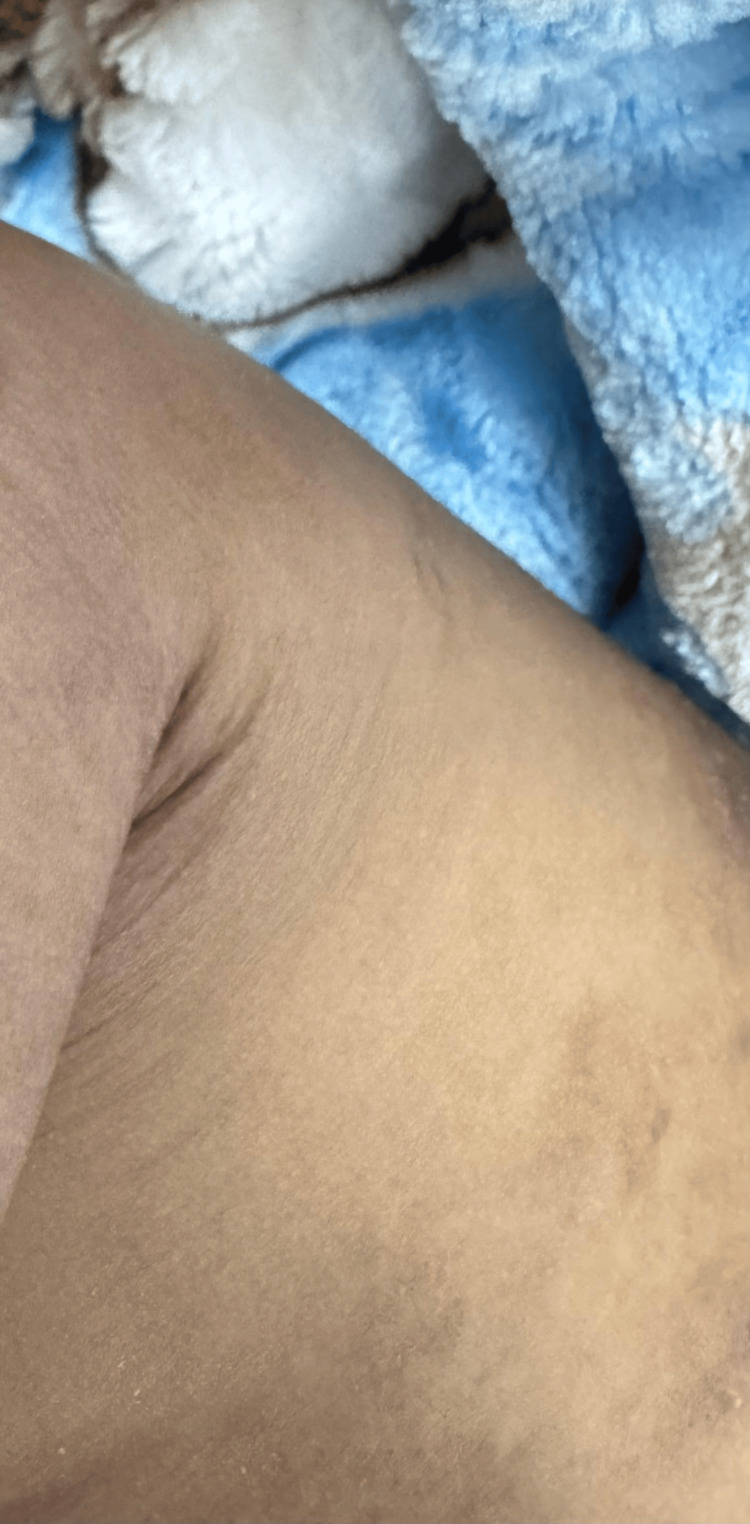
Cutaneous lesions involving the upper extremities consistent with eczematous dermatitis, a recognized feature in patients with ENaC dysfunction ENaC: epithelial sodium channel.

During this period, the infant developed erythematous skin lesions over the face, trunk, and upper arms (Figures 1, 2). These cutaneous findings may serve as an additional clinical clue supporting the diagnosis of systemic pseudohypoaldosteronism type 1. Dermatology evaluation diagnosed atopic dermatitis, for which topical zinc oxide, fusidic acid, and emollients were prescribed.

Hormonal evaluation demonstrated normal levels of 17-hydroxyprogesterone, adrenocorticotropic hormone, and cortisol, effectively excluding congenital adrenal hyperplasia. In contrast, plasma renin and aldosterone levels were markedly elevated (Table 1). These findings demonstrate elevated renin and aldosterone levels in the presence of hyponatremia and hyperkalemia, consistent with aldosterone resistance.

**Table 1:** Hormonal assay results ACTH: adrenocorticotropic hormone.

Laboratory Test	Patient’s Value	Normal Range
ACTH	15.9 pg/mL	9-152 pg/mL
Cortisol	142.70 nmol/L	31.45-519 nmol/L
17-hydroxyprogesterone	5.2 ng/mL	0.7-9 ng/mL
Aldosterone	289 ng/dL	5-132 ng/dL
Renin	7,700 ng/L	6.3-149 ng/L

Key diagnostic clues in this case included persistent severe hyperkalemia and hyponatremia from early infancy, lack of response to fludrocortisone therapy, markedly elevated renin and aldosterone levels, and recurrent metabolic decompensation despite appropriate management. Based on these findings, pseudohypoaldosteronism was strongly suspected. Genetic analysis using whole-exome sequencing identified a homozygous nonsense variant in the SCNN1B gene (Table 2), confirming the diagnosis of systemic pseudohypoaldosteronism type 1. This finding explains the underlying defect in epithelial sodium channel function and accounts for the observed clinical phenotype. This variant has not been previously reported in the literature and is considered novel.

**Table 2:** Genetic variant identified by whole-exome sequencing PolyPhen (Harvard Medical School, Boston, MA, USA). SNP: single-nucleotide polymorphism, N/A: not applicable, PolyPhen: Polymorphism Phenotyping, GVGD: Grantham variation and Grantham deviation, SIFT: Sorting Intolerant From Tolerant, Conservation_nt: nucleotide conservation, aa: amino acid.

Gene	Variant Coordinates	Amino Acid Change	SNP Identifier	Zygosity	In Silico Parameters	Type and Classification
SCNN1B	NM_000336.2:c.1573C>T	p.(Gln525*)	N/A	Homozygous	PolyPhen: N/A. Align-GVGD: N/A. SIFT: N/A. MutationTaster: N/A. Conservation_nt: high. Conservation: aa.	Likely pathogenic

Management included initiation of oral sodium chloride supplementation, gradually increased to 20 mmol/kg/day, along with sodium bicarbonate therapy, resulting in stabilization of electrolyte and acid-base status. Additional potassium-lowering strategies included oral calcium polystyrene sulfonate and a potassium-restricted renal formula. The patient required a prolonged NICU stay of approximately four months and was managed by a multidisciplinary team including neonatology, pediatric endocrinology, nephrology, infectious diseases, and dermatology. Following discharge, he experienced multiple emergency visits and pediatric intensive care admissions due to recurrent episodes of hyperkalemia, hyponatremia, metabolic acidosis, and intercurrent infections, including *Klebsiella* sepsis. A nasogastric tube was inserted to facilitate medication administration, and gastrostomy placemen was discussed with the family.

Despite recurrent metabolic crises and repeated hospitalizations, the infant demonstrated satisfactory growth and acceptable developmental progress for his age. At eight months, his weight was 8 kg and length was 78 cm, both corresponding to approximately the 25th percentile on CDC growth charts. Developmentally, he was able to sit with support and produced multiple vocal sounds appropriate for his age.

## Discussion

Systemic pseudohypoaldosteronism type 1 is a rare but potentially life-threatening disorder resulting from resistance to aldosterone at the epithelial sodium channel level. In contrast to the renal-limited form, systemic PHA1 involves multiple organs due to widespread expression of ENaC in epithelial tissues, including the kidney, colon, lungs, sweat glands, and salivary glands [1,3]. Dysfunction of ENaC leads to impaired sodium reabsorption and reduced potassium excretion in the distal nephron, resulting in persistent hyperkalemia, hyponatremia, and metabolic acidosis.

The clinical presentation in our patient, severe neonatal hyperkalemia, hyponatremia, and elevated renin and aldosterone levels, is consistent with previously reported cases of systemic PHA1 [4]. Because these biochemical abnormalities closely resemble those seen in congenital adrenal hyperplasia, many patients initially undergo evaluation for adrenal disorders. Normal cortisol and 17-hydroxyprogesterone levels in our patient helped exclude adrenal insufficiency, supporting the diagnosis of aldosterone resistance.

Mutations affecting the β-subunit of ENaC encoded by the SCNN1B gene have been reported in several patients with systemic PHA1 and are associated with severe electrolyte disturbances during infancy [5]. Truncating variants such as nonsense mutations frequently result in complete loss of channel function and are typically associated with more severe clinical manifestations. In our patient, whole-exome sequencing identified a homozygous nonsense variant resulting in a premature stop codon, which likely leads to a nonfunctional β-subunit and impaired ENaC activity.

Management of systemic PHA1 is challenging and requires lifelong therapy focused on maintaining electrolyte balance. Treatment usually includes high-dose sodium supplementation, correction of metabolic acidosis, and pharmacologic strategies to control hyperkalemia [6]. Potassium-binding agents such as calcium polystyrene sulfonate are commonly used to enhance gastrointestinal potassium elimination. In addition, potassium-restricted formulas may help reduce dietary potassium intake in infants with recurrent hyperkalemia.

The prognosis of systemic pseudohypoaldosteronism type 1 is variable and largely depends on the severity of electrolyte disturbances and the frequency of intercurrent illnesses. Affected infants often experience recurrent life-threatening episodes of hyperkalemia, hyponatremia, and metabolic acidosis, particularly during early childhood, necessitating repeated hospitalizations and intensive medical management. With advancing age, some patients may demonstrate partial clinical improvement, possibly due to maturation of renal tubular function and compensatory mechanisms in sodium handling. However, many patients require long-term sodium supplementation and ongoing monitoring to prevent metabolic decompensation. Early recognition and appropriate management are essential to reduce morbidity and improve overall outcomes [2]. In our patient, the recurrent metabolic crises and repeated hospitalizations during infancy highlight the severe clinical course associated with truncating SCNN1B variants.

Intercurrent illnesses such as infections or dehydration may precipitate acute salt-wasting crises requiring hospitalization and intensive electrolyte management. Several reports have documented repeated hospital admissions in affected infants due to recurrent hyperkalemia and metabolic decompensation during early childhood [7]. With age, some patients experience partial clinical improvement, possibly related to maturation of renal tubular function and adaptive mechanisms in sodium transport.

Cutaneous manifestations may also occur in systemic PHA1 due to impaired sodium reabsorption in sweat ducts. Elevated sodium concentration in sweat may cause skin irritation and eczematous dermatitis, which has been reported in several patients with ENaC mutations [8]. These dermatologic findings provide an additional clinical clue that may support the diagnosis.

Genetic testing plays a crucial role in confirming the diagnosis of systemic PHA1 and distinguishing it from other salt-wasting disorders. Identification of the causative variant not only confirms the diagnosis but also facilitates genetic counseling for families, particularly in populations with high rates of consanguinity [9]. Genetic testing should be considered in selected cases with persistent or unexplained electrolyte abnormalities. Our case expands the mutational spectrum of SCNN1B-related PHA1 and emphasizes the importance of considering this condition in neonates presenting with persistent hyperkalemia and hyponatremia despite normal adrenal hormone levels.

## Conclusions

Systemic pseudohypoaldosteronism type 1B (PHA1B) is a rare but serious neonatal salt-wasting disorder that can easily be mistaken for other endocrine or renal conditions, particularly congenital adrenal hyperplasia. This case highlights the importance of considering PHA1B in any neonate presenting with persistent hyponatremia, hyperkalemia, and metabolic acidosis despite normal adrenal hormone levels and appropriate medical therapy. The identification of a novel SCNN1B variant in this Saudi infant expands the mutational spectrum associated with this condition and underscores the value of early genetic testing, especially in populations with high rates of consanguinity. Early molecular diagnosis allows accurate counseling of families and may guide long-term management strategies. Prompt diagnosis, aggressive electrolyte management, and multidisciplinary care are essential to prevent life-threatening complications, reduce recurrent hospitalizations, and improve long-term outcomes in affected infants. This case underscores the importance of recognizing key diagnostic features and highlights the role of genetic confirmation in guiding management, anticipating disease severity, and providing appropriate genetic counseling.
